# Cell free fetal DNA testing in maternal blood of Romanian pregnant women

**Published:** 2015-10

**Authors:** Viorica E Radoi, Camil L Bohiltea, Roxana E Bohiltea, Dragos N Albu

**Affiliations:** 1 *Department of Medical Genetics, UMF Carol Davila, Romania, Bucharest.*; 2 *Department of Maternal and Fetal Medicine, Medlife Romania, Bucharest.*

**Keywords:** Prenatal, Fetal, DNA

## Abstract

**Background:**

The discovery of circulating fetal DNA in maternal blood led to the discovery of new strategies to perform noninvasive testing for prenatal diagnosis.

**Objective:**

The purpose of the study was to detect fetal aneuploidy at chromosomes 13, 18, 21, X, and Y by analysis of fetal cell-free DNA from maternal blood, without endangering pregnancy.

**Materials and Methods:**

This retrospective study has been performed in Bucharest at Medlife Maternal and Fetal Medicine Department between 2013-2014. In total 201 women were offered noninvasive prenatal test. Maternal plasma samples were collected from women at greater than 9 weeks of gestation after informed consent and genetics counseling.

**Results:**

From 201 patients; 28 (13.93%) had screening test with high risk for trisomy 21, 116 (57.71%) had advanced maternal age, 1 (0.49%) had second trimester ultrasound markers and the remaining 56 patients (27.86%) performed the test on request. Of those patients, 189 (94.02%) had a “low risk” result (<1/10,000). Of those who had a low risk result, 2 continued on to have amniocentesis with normal results.Five patients (2.48%) received “high risk” results (>99% risk) all for trisomy 21 (T21). T21 was confirmed by amniocentesis in 1 patient and the other 4 patients declined confirmation. The 7 remaining patients (3.48%) had a low fetal fraction of DNA.

**Conclusion:**

It is probably that prenatal diagnosis using fetal DNA in maternal blood would play an increasingly role in the future practice of prenatal testing because of high accuracy.

## Introduction

Prenatal screening and diagnosis are frequently used in antenatal care and are considered to be a very useful tool for physicians and women to take informed decision about the continuation of pregnancies affected by genetics diseases. Diagnostic testing requires removal of a sample of fetal cells from chorionic villus (between 11-13 weeks) or amniotic fluid (after 16 weeks of gestation). These invasive procedures have a risk of miscarriage between 0.5-2% (1).

The purpose of prenatal diagnosis is to provide reliable clinical information to parents on the risk of having babies affected by genetics disorders. Placenta is the central location of an important bidirectional transfer but is not a barrier between the pregnant woman and her genetically different fetus (2). The discovery of circulating fetal DNA in maternal blood led to the development of new strategies to perform noninvasive testing for prenatal diagnosis, without any complication for the fetus and pregnancy. The possibility to measure rapidly and reproducibly fetal cell-free DNA with noninvasive techniques has many clinical applications including gender detection, diagnosis of fetal aneuploidy such as 13, 18 and 21 trisomies, complications of pregnancy, X-linked diseases, cystic fibrosis, and several other monogenic diseases (3-6).

Plasma DNA obtained from a pregnant woman contains both maternal and fetal DNA. The fetal DNA proportion in maternal plasma is relatively consistent as determined using polymorphic genetics markers across different chromosomes in euploid pregnancies. For aneuploid pregnancies, the proportion of observed fetal DNA measured using polymorphic genetics markers for the aneuploid chromosome would be modified (7, 8)

Abnormal fetal DNA concentrations in maternal plasma have been found in other pregnancy-associated complications, such as preterm labor and preeclampsia, as well as in pregnancies complicated by fetal trisomy 21 (8, 9) after delivery, fetal DNA is cleared rapidly from maternal plasma, with a half-life in the order of minutes. This clearance kinetics has a different pattern from fetal cell clearance, where long-term persistence has been observed (7)

The purpose of this study was to detect fetal aneuploidy at chromosomes 13, 18, 21, X, and Y by analysis of fetal cell-free DNA from maternal blood, without endangering pregnancy.

## Materials and methods

This retrospective study has been performed in Bucharest at Medlife Maternal and Fetal Medicine Department between January 2013-2014. In our department, non-invasive prenatal testing is offered for various indications including positive aneuploidy serum screening (double test, triple test, quad test, cut off >1/250), advanced maternal age, abnormal ultrasound markers (increased nuchal translucency in the first trimester, echogenic bowel, echogenic focus in the heart, and choroid plexus cyst in the second trimester) and to those who request a discussion of prenatal testing options including invasive procedures. On week 9 the test was performed by request of the patients. Patients receive genetics counseling which contains information about the fetal cell-free DNA technology, and specifies the screening nature of this testing and limitation of screening to aneuploidy involving chromosomes 21, 18, and 13. In total 201 women were offered non-invasive prenatal test. Maternal plasma samples were collected from patients at greater than 9 weeks of gestation after informed consent.

The method targets 19500 single-nucleotide polymorphisms (SNPs) in a single multiplex PCR reaction, thus can differentiate between maternal and fetal genotypes and uses the Next-generation Aneuploidy Test Using SNPs (NATUS) algorithm (Patented by Natera Laboratory) to analyze data. Panorama test utilizes the mother’s white blood cells to isolate and identify her DNA, and then uses this information to “extract” the maternal genotype, resulting in a more accurate fetal genotype. Panorama test has a unique bioinformatics approach, results in greater quality control capabilities.

The goal of this report is to share our experience, highlighting the clinical utility and limitations of this novel screening test.

Informed consent was obtained from all human adult participants. The study was approved by the Ethics Committee of Life Memorial hospital.

**Statistical analysis**

Descriptive data are presented in numbers and percentages for categorical variables using PROC FREQ software.

## Results

Demographic maternal characteristics of women undergoing the test are presented in table I. The median maternal age of women was 36.8 years (range 24-44 years) and the median gestational age at sampling was 15 weeks + 1 day (range 9 weeks + 1 day-28 weeks).

**Figure 1:**
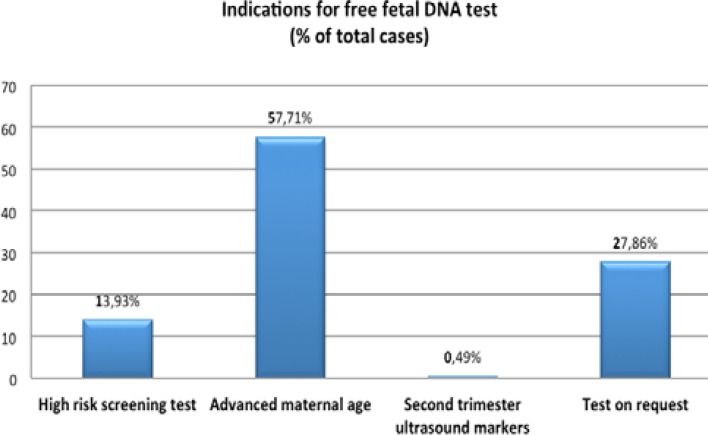
The graphic shows the indications of the free fetal DNA testing for aneuploidies of the study group.

The test was accepted by 201 patients (99.5%). From 201 patients who accepted the test 28 (13.93%) had screening test with high risk for trisomy 21 (cut off >1/250), 116 (57.71%) had advanced maternal age (over 35 years), 1 (0.49%) had second trimester ultrasound markers and the remaining 56 patients (27.86%) performed the test on request (no medical reason) (Figure 1). Of those patients who accepted testing, 189 (94.02%) had a “low risk” result (<1/10,000). Of those who had a low risk result, 2 continued on to have amniocentesis with normal results. 

**Figure 2:**
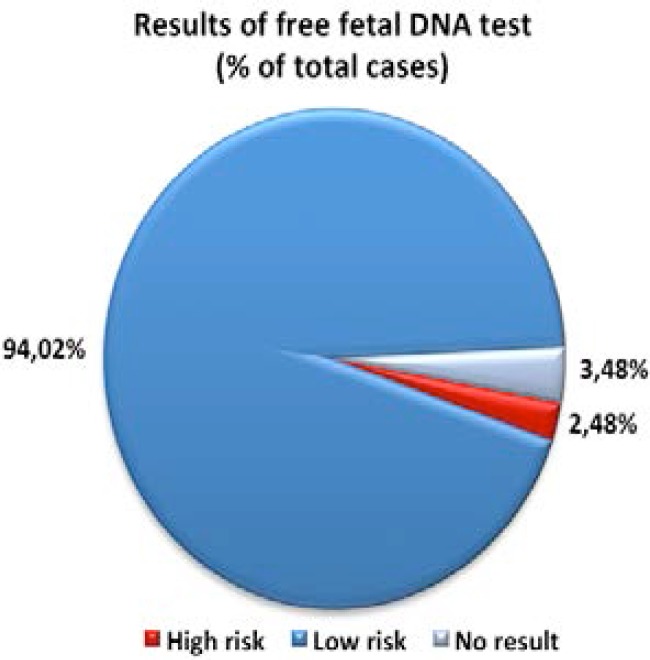
The graphic shows the results of the test for patients in this study 94.02% with low risk, 2.48% with high risk and 3.48% with no result

Five patients (2.48%) received “high risk” results (>99% risk) all for trisomy 21 (T21). T21 was confirmed by amniocentesis in 1 patient and the other 4 patients declined confirmation but are using this information for delivery management (made abortion). The 7 remaining patients (3.48%) received “no result” reports because of a low fetal fraction of DNA. In group with “no result” maternal weight was higher but the difference was not significant (maybe because of small lot of patients) (Figure 2).

**Table I:** Demographic maternal characteristicsof participants

**Characteristics**	**Values **
Maternal age (years)	36.8 (24-44)
Gestational age (weeks)	15.1 (9.1-28)
Maternal weight (kg)	64.9 (45-114)
Ethnicity (%)	
Caucasian	195 (97.01%)
Asian	6 (2.98%)

Descriptive data are presented in n (%) for categorical variables. The maternal age, gestational age and maternal weight is presented as mean (range) value.

## Discussion

The 1997 discovery of free fetal DNA in maternal plasma initiated clinical researchers’ efforts to discover a precise method for non- invasive prenatal testing for fetal genetics conditions. Different methods have recently been developed as highly accurate noninvasive screening tools for common fetal chromosomal aneuploidies. Incorporating these noninvasive techniques into clinical practice will impact the current prenatal screening algorithm for fetal aneuploidy, in which genetics counseling plays an integral role.

As shown in our study, the majority of pregnancies identified by the combined test as being at high risk for trisomy 21, 18, or 13 are euploid. The use of fetal cell-free DNA testing would reduce the number of unnecessary invasive test (amniocentesis and chorionic villi sampling) and eliminate their associated risk of miscarriage. These data are consistent with those obtained by Nicolaides and colleagues (10). Given non-invasive prenatal testing’s important superior sensitivity and specificity compared to other available aneuploidy screening such as, first trimester nuchal translucency and/or biochemical screening and second trimester triple/quad screening, it is important that patients understand the significant implication of a positive result prior to undergoing testing.

We find that non-invasive prenatal testing is chosen by many women in place of diagnostic testing. It is essential that patients have genetics counseling to understand the limitation of this technology which is a screening test. Patients who have other factors suggestive of a chromosomal abnormality (such as abnormal ultrasound or parental structural chromosomal abnormalities) should receive genetics counseling and have the option of conventional confirmatory diagnostic testing, regardless of non-invasive test results, because this test does not screen for all chromosomal or genetics conditions and maternal abnormalities, such as maternal mosaicism. In addition it can contribute to false positive results with NIPT tests that cannot distinguish between fetal and maternal DNA. In fact, maternal mosaicism led to 5-9% of false positive results when screening sex chromosomes with NIPT tests (11).

The primary source of non-maternal cell free DNA in the maternal circulation is thought to be apoptosis of placental cells (syncytiotrophoblast), while maternal hematopoietic cells are the source of most maternal cell free DNA. Somatic mosaicism, placental mosaicism and maternal CNV changes can modify the accuracy of cell free DNA testing in maternal blood (12).

Approximately 5% of cases failed to meet quality control metrics and a portion of cell free DNA samples will not return a result because of a low fetal fraction. Recent data suggests that this “no-call” group is at increased risk of chromosomal aneuploidy (12). Diagnostic testing and/or repeat cell free DNA should be strongly considered for such patients.

Counseling regarding the limitations of cell free fetal DNA testing should include a discussion that the screening test provides information regarding only trisomy 21, trisomy 18 and trisomy 13. Other limitations of cell free fetal DNA includes the lack of outcome data for low-risk populations; therefore, cell free fetal DNA testing is not currently recommended for low-risk women. Preliminary data available on twins demonstrate accuracy in a very small cohort, but more information is needed before use of this test can be recommended in multiple gestations (13, 14)

## Conclusion

It is possible that non-invasive prenatal diagnosis using fetal DNA in maternal blood would play an increasingly important role in the future practice of prenatal testing. However it is important to address the ethical, legal and social issues surrounding such developments. The positive side of non-invasive testing is the avoidance to harm the fetus that would be associated with invasive testing. It does not replace the precision obtained with diagnostic tests, such as chorionic villus sampling (CVS) or amniocentesis, and currently does not offer other genetics information.
